# Neoadjuvant immunotherapy for lung cancer: current limitations and future prospects

**DOI:** 10.3389/fimmu.2026.1752298

**Published:** 2026-01-28

**Authors:** Kunpeng Yang, Lei Wang, Zhe Wang, Peiyun Lv, Chenglun Cai, Hui Zhao, Wenjuan Sun, Bao Wang

**Affiliations:** 1 Department of Thoracic Oncology Surgery II, Jilin Cancer Hospital, Changchun, Jilin, China; 2 School of Clinical Medicine, Changchun University of Chinese Medicine, Changchun, China

**Keywords:** biomarkers, neoadjuvant immunotherapy for lung cancer, non-small cell lung cancer, pathological response, personalized treatment

## Abstract

Lung cancer is the leading cause of cancer-related mortality globally, with non-small cell lung cancer (NSCLC) accounting for the majority of cases. For resectable early-stage NSCLC, surgery and adjuvant chemotherapy remain the standard treatments, but the recurrence rate is high, and long-term survival outcomes are suboptimal. In recent years, immunotherapy, particularly immune checkpoint inhibitors (ICIs), has revolutionized the treatment of advanced NSCLC and is gradually being extended to early-stage disease. Neoadjuvant immunotherapy, as an emerging strategy, aims to activate anti-tumor immune responses preoperatively, eliminate microscopic metastases, and downstage tumors, thereby improving surgical resectability and enhancing long-term survival for patients. Several clinical trials have demonstrated that neoadjuvant immunotherapy, either alone or in combination with chemotherapy, significantly increases the major pathological response (MPR) and pathological complete response (pCR) rates, translating into improvements in event-free survival (EFS). However, this promising therapeutic approach faces numerous challenges, including the lack of precise biomarkers for efficacy prediction, unclear treatment strategies for patients with driver gene-positive tumors, primary and secondary resistance, management of immune-related adverse events (irAEs), and the complexities of post-treatment efficacy evaluation. This review aims to comprehensively summarize the latest clinical evidence on neoadjuvant immunotherapy for lung cancer, delve into the main challenges and opportunities encountered in clinical practice, and explore its future directions, including novel combination therapies, personalized treatment strategies, and the application of innovative technologies, with the goal of optimizing clinical management for early-stage lung cancer patients and advancing research in this field.

## 1 Introduction

Lung cancer is one of the most common and deadly malignancies worldwide, posing a significant threat to human health (1). In China, lung cancer also ranks as a leading cancer type, characterized by high incidence and mortality rates (2). Non-small cell lung cancer (NSCLC) accounts for approximately 85% of all lung cancer cases, with about 20-25% of patients diagnosed with resectable early-stage (stage I-IIIA) disease (3). For these patients, radical surgical resection remains the primary treatment modality, with the goal of achieving a cure (4). However, despite complete surgical resection, a substantial number of patients experience treatment failure due to postoperative recurrence and distant metastasis, resulting in suboptimal five-year survival rates (5).

To improve prognosis, adjuvant chemotherapy has become the standard treatment for certain high-risk patients; however, its survival benefit is limited, with a modest five-year survival rate improvement of only 4-5% (6). Therefore, the development of more effective perioperative systemic treatment strategies to reduce the risk of recurrence and improve cure rates remains a critical issue in the field of lung cancer.

In recent years, the advent of immune checkpoint inhibitors (ICIs), particularly antibodies targeting programmed cell death protein 1 (PD-1) and its ligand (PD-L1), has significantly altered the treatment paradigm for advanced NSCLC (7). These drugs block tumor cells’ immune evasion pathways and re-activate the body’s anti-tumor immune response, offering patients prolonged survival benefits. Given their significant success in advanced disease, researchers have begun exploring the application of immunotherapy in early-stage, potentially curable NSCLC patients, with neoadjuvant treatment gaining increasing attention (8).

**Figure 1:**
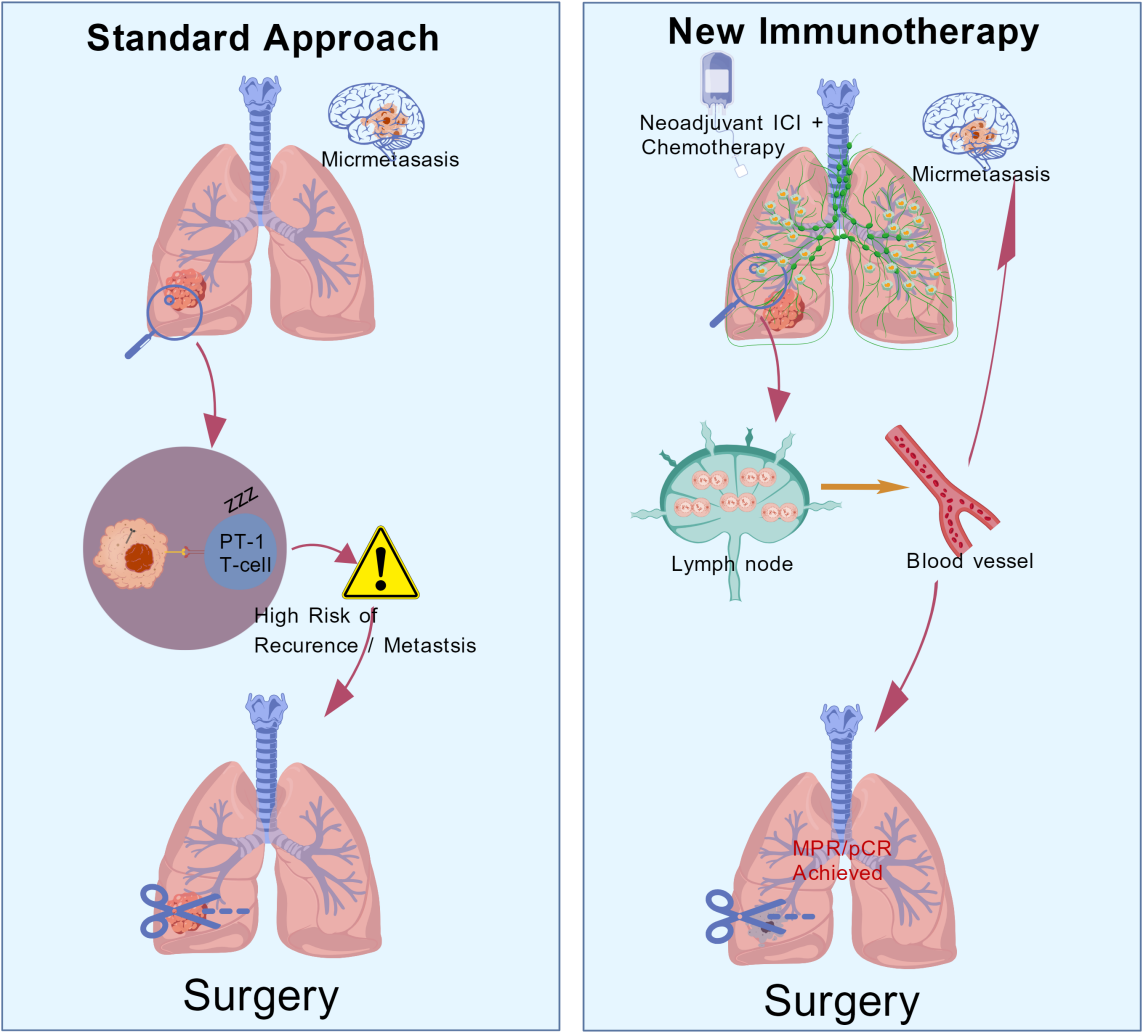
Mechanisms and theoretical advantages of neoadjuvant immunotherapy. This schematic elucidates the fundamental immunological disparities between neoadjuvant and adjuvant therapies, with a specific focus on the unique value of the “therapeutic window.” In the neoadjuvant setting (right panel), the primary tumor and intact draining lymph nodes serve as a rich repository of macroscopic tumor antigens. Immunotherapy via checkpoint inhibition (ICI) maximizes the priming and expansion of polyclonal tumor-specific T cells. Subsequently, these activated T cells enter the systemic circulation to prospectively identify and eradicate systemic micrometastases prior to surgical intervention. Conversely, in the traditional surgical or adjuvant therapy model (left panel), the breadth and durability of the immune response may be compromised. This limitation arises because the surgical procedure often removes draining lymph nodes beforehand, leaving only minimal residual antigens in the post-operative state. Overall, the figure visualizes how neoadjuvant intervention leverages the intact immune anatomy to maximize the T-cell priming effect.

Compared with adjuvant therapy, neoadjuvant immunotherapy offers distinct theoretical advantages (**Figure 1**). By administering treatment while the primary tumor and draining lymph nodes are intact, neoadjuvant therapy can utilize the full tumor antigen repertoire to maximally prime tumor-specific T cells, potentially eliminating micro-metastatic lesions more effectively than postoperative strategies (9). Furthermore, pathological assessment of the resected specimen provides direct feedback on tumor sensitivity, guiding subsequent adjuvant management (10).

Building on these advantages, numerous global clinical trials on neoadjuvant immunotherapy for NSCLC have been conducted, yielding encouraging results that are driving transformative changes in clinical practice (11). However, as its application deepens, neoadjuvant immunotherapy has also revealed numerous challenges and unresolved questions. The core aim of this article is to systematically review the current clinical evidence on neoadjuvant immunotherapy for lung cancer, thoroughly analyze the limitations and challenges it faces, and explore future directions and potential opportunities, in order to provide theoretical support and practical guidance for maximizing the clinical value of this innovative therapy.

## 2 Current evidence

In recent years, several key clinical studies have greatly expanded our understanding of the efficacy and safety of neoadjuvant immunotherapy for NSCLC, gradually establishing its important role in the treatment of resectable NSCLC.

Among these studies, the CheckMate-816 trial stands as a milestone Phase III randomized controlled trial in this field. This study compared neoadjuvant nivolumab in combination with platinum-based chemotherapy to chemotherapy alone in patients with resectable stage IB–IIIA NSCLC. The results demonstrated that the combination therapy group had a significantly higher pathological complete response (pCR) rate than the chemotherapy-only group (24.0% vs 2.2%), and event-free survival (EFS) was also significantly improved (12). Based on these findings, nivolumab combined with chemotherapy became the first FDA-approved neoadjuvant immunotherapy regimen for resectable NSCLC (13). In addition to this study, several other Phase II trials have shown similarly positive trends. For example, the SAKK 16/14 trial administered durvalumab sequentially after cisplatin/docetaxel chemotherapy, with continued maintenance therapy post-surgery, achieving a 1-year EFS rate of 73% and a major pathological response (MPR) rate of 62% (14). Furthermore, a meta-analysis of nine randomized controlled trials involving 3431 patients confirmed that immunotherapy combined with chemotherapy significantly outperformed chemotherapy alone in terms of EFS, pCR, MPR, surgical resection rates, and R0 resection rates (15). These results provide strong evidence for the broader adoption of neoadjuvant immunotherapy in clinical practice.

Regarding efficacy evaluation, MPR and pCR have become widely used surrogate endpoints in clinical trials of neoadjuvant therapy. The scientific basis for this is that the degree of tumor regression induced by preoperative treatment can indirectly reflect the clearance of micrometastases, thereby predicting long-term survival benefits (16). Numerous studies have shown that patients who achieve MPR or pCR have significantly better EFS and overall survival (OS) compared to those who do not (17). However, standardizing the pathological assessment of these responses remains a key challenge in clinical practice. The International Association for the Study of Lung Cancer (IASLC) has issued multidisciplinary expert recommendations advocating for systematic quantitative evaluation of resected specimens, calculating the proportions of viable tumor cells, necrosis, and stromal components (including inflammation and fibrosis), to improve consistency and comparability of assessments (18). Nevertheless, tumors treated with immunotherapy often exhibit substantial infiltration of inflammatory cells and fibrotic responses, making it challenging to delineate the boundaries of residual tumor tissue, which remains somewhat subjective and technically difficult (19).

Mechanistically, high-throughput technologies such as single-cell RNA sequencing have validated the biological impact of neoadjuvant immunotherapy. Studies reveal that patients achieving MPR exhibit distinct features, including the upregulation of MHC-II-mediated antigen presentation in tumor cells and a significant expansion of cytotoxic T cells and NK cells, alongside a marked reduction in immunosuppressive Tregs (20). These findings confirm that neoadjuvant therapy effectively reshapes the tumor immune microenvironment (TIME), converting “cold” tumors to “hot” ones and providing a biological basis for the observed clinical efficacy (21).

Safety and surgical feasibility are key concerns for clinicians when adopting neoadjuvant immunotherapy. Overall, the available research indicates that the safety profile of immunotherapy combined with chemotherapy is manageable. Although the incidence of grade 3 or higher adverse events is slightly higher compared to chemotherapy alone, most toxic events can be effectively controlled with appropriate management, without significantly increasing treatment-related mortality (15). Immune-related adverse events (irAEs), specific to ICIs, require recognition and management according to established guidelines to ensure patient safety (22). In terms of surgical feasibility, the majority of studies report that neoadjuvant immunotherapy does not lead to delays in surgery or an increased rate of surgery cancellations (23). However, some surgical teams have observed that immunotherapy may induce fibrosis and adhesion in surrounding tumor tissues, thereby increasing the difficulty of surgery and the need for thoracotomy (24). This not only represents a clinical management challenge but also presents a critical opportunity for translational research. This observation suggests that new imaging techniques (e.g., AI-based CT texture analysis) or serum biomarkers may be useful for predicting the degree of fibrosis preoperatively, helping surgeons plan their procedures. Additionally, further research into the cellular and molecular mechanisms of this fibrosis (whether it is “beneficial” inflammatory repair or “detrimental” tissue remodeling) is a key translational research direction. Therefore, preoperative assessment and communication by a multidisciplinary team with extensive experience, along with enhanced basic and clinical cross-collaboration, is crucial for optimizing perioperative management and ensuring patient safety.

In conclusion, existing evidence strongly supports the clinical value of neoadjuvant immunotherapy, which not only significantly improves pathological response rates and EFS but also enhances long-term outcomes for resectable NSCLC patients without substantially increasing surgical risks. This strategy’s clinical value is gradually being confirmed, providing a solid foundation for future research and practice, and paving the way for innovative changes in the comprehensive treatment landscape of lung cancer.

Although landmark trials have solidified the standing of neoadjuvant immunotherapy, underlying inconsistencies remain regarding the precise selection of beneficiary populations and the standards for efficacy assessment. Consequently, a more critical scrutiny of emerging biomarkers and pathological surrogate endpoints is warranted.

## 3 Beyond PD-L1: a critical comparison and validation gaps of emerging biomarkers

### 3.1 Circulating tumor DNA: from static stratification to dynamic monitoring

Functioning as a non-invasive liquid biopsy tool, ctDNA enables the assessment of treatment response earlier than traditional imaging through the monitoring of “molecular clearance.” Furthermore, it has been identified as the most robust independent predictor of postoperative recurrence risk. Regarding clinical feasibility, although ctDNA facilitates the capture of tumor clonal evolution, significant challenges persist in early-stage (Stage I) NSCLC. Due to minimal tumor DNA shedding, detection is constrained by severe sensitivity bottlenecks and high false-negative rates. Additionally, interference from clonal hematopoiesis of indeterminate potential (CHIP) frequently results in false-positive findings, thereby escalating both interpretative complexity and associated costs (25).

A critical validation gap remains: prospective interventional trials have yet to demonstrate that adjusting postoperative strategies based on ctDNA findings translates into definitive survival benefits. Furthermore, a consensus regarding the standardization of detection assays—specifically between tumor-informed and agnostic approaches—has not yet been reached within the field (26).

### 3.2 Antigen presentation mechanism signatures: unveiling the root of “immune evasion”

APM signatures, characterized by expression markers such as NLRC5 and TAP1, evaluate the capacity of MHC molecules to present neoantigens to T cells. These signatures elucidate the underlying biological mechanisms governing primary resistance to immunotherapy, even within distinct subsets of “hot” tumors (27). Although APM scores have been verified as predictors of therapeutic efficacy independent of tumor mutational burden (TMB)—with elevated scores correlating with significantly improved prognosis—clinical implementation remains restricted. The reliance on high-quality RNA sequencing or complex multiplex immunohistochemistry (mIHC) poses a substantial barrier to widespread adoption in routine pathological practice (28).

Significant validation gaps persist within this domain, most notably the absence of standardized scoring thresholds (cutoffs) and the lack of prospective efficacy validation within large-scale neoadjuvant cohorts (29).

### 3.3 Tumor immune microenvironment and spatial analysis: location dictates fate

The analysis of the TIME via multiplex immunofluorescence (mIF) and spatial transcriptomics facilitates the identification of spatial architectures, such as tertiary lymphoid structures (TLS). Furthermore, metrics quantifying the physical distance between CD8+ T cells and tumor cells—known as proximity scores—have been shown to provide predictive value superior to simple cellular quantification (30, 31). Nevertheless, the implementation of these high-throughput spatial analysis techniques is currently hindered by significant barriers, including high technical thresholds, prohibitive costs, and poor data reproducibility across divergent experimental platforms. Consequently, their application remains largely confined to translational research.

Critical future gaps involve the establishment of standardized digital pathology workflows and the demonstration of the cost-effectiveness of these complex spatial metrics in guiding clinical strategies for treatment de-escalation or intensification. To provide a direct comparison of the clinical utility of these biomarkers, a critical summary of their predictive targets, clinical feasibility, core limitations, and validation gaps is presented in **Table 1**.

**Table 1:** Critical comparison of emerging biomarkers in neoadjuvant NSCLC treatment.

Biomarker	Predictive target	Clinical feasibility (Current)	Key limitations & validation gaps
ctDNA Dynamic Monitoring	MRD/Therapeutic Response Kinetics	Intermediate (Expanding adoption)	Low sensitivity in early-stage lung cancer; lack of Level I evidence for survival benefits from ctDNA-guided interventions; lack of consensus on assay standardization (Tumor-informed vs. Agnostic).
APM Gene Signatures	Tumor “Visibility”/Immune Evasion	Low (Predominantly research-based)	Reliance on RNA-seq or multiplex IHC (mIHC); absence of standardized scoring systems and cutoff values; insufficient validation in large-scale early-stage lung cancer cohorts.
TIME/Spatial Analysis (mIF)	Spatial Immune Cell Interactions/TLS	Low (High technical threshold)	Complexity of data analysis; lack of cross-platform reproducibility; undefined cost-effectiveness.
PD-L1 (IHC)	Immunosuppressive Potential	High (Gold Standard)	Represents a static snapshot incapable of reflecting dynamic changes; spatial heterogeneity; poor negative predictive value (NPV).

## 4 Revisiting pathological endpoints: dilemmas in surrogacy, heterogeneity, and standardization

### 4.1 Limitations of surrogate endpoints: correlation ≠ causality

Meta-analyses indicate a significant correlation between patients achieving MPR or pCR and improvements in EFS and OS at the individual level (e.g., an EFS hazard ratio of approximately 0.49 for pCR patients). However, at the trial level, an increase in pCR rates does not consistently translate linearly into OS benefits (32). This discrepancy may stem from the dilution of OS differences by subsequent second-line therapies, such as antibody-drug conjugates (ADCs) or targeted treatments. Additionally, long-term toxicities associated with immunotherapy—including pneumonitis sequelae and endocrine disorders—may partially offset the survival advantages conferred by tumor control (33). Therefore, scientific prudence is required when considering pathological remission as a definitive surrogate endpoint for long-term survival.

### 4.2 Inter-observer variability and assessment challenges

Tumor regression induced by immunotherapy exhibits a distinct “immune clearance” pattern, characterized by extensive proliferative fibrosis, neovascularization, and dense immune cell infiltration. These features impose significant subjective challenges for pathologists in delineating the original “tumor bed,” thereby directly impacting the accurate quantification of residual viable tumor cells (34). Research indicates discrepancies in pathological assessment consistency between squamous cell carcinoma and adenocarcinoma. Furthermore, in real-world practice, the absence of a unified, standardized protocol for extensive sampling of the entire tumor bed in large masses frequently results in the overestimation or underestimation of pCR rates (35). This variability underscores the urgent need to implement the standardized assessment guidelines recommended by the IASLC and to integrate AI-assisted analysis in future practice.

### 4.3 The “survival-pathology” dissociation in molecular subgroups

A distinct dissociation, or “decoupling,” between pathological response and survival prognosis is observed within specific driver gene-positive and co-mutant populations. For instance, patients with EGFR mutations undergoing neoadjuvant targeted therapy often achieve significant EFS extension despite extremely low pCR rates (typically <10%). This suggests that pCR may not be the optimal endpoint for agents that function primarily by inhibiting proliferation rather than inducing acute necrosis (34). Conversely, patients harboring STK11/KEAP1 co-mutations maintain a high risk of postoperative recurrence due to aggressive biological behavior and inherent immune resistance, even when partial pathological remission is observed (36). The existence of these specific subgroups demonstrates that morphological assessment alone cannot fully capture the molecular heterogeneity and malignant potential of the tumor.

## 5 Clinical challenges and opportunities

Despite the groundbreaking progress made in the treatment of resectable NSCLC with neoadjuvant immunotherapy, several challenges remain in the process of its widespread adoption into routine clinical practice. Overcoming these limitations and optimizing the strategy is a central issue that future research must address.

First, accurately selecting the patients most likely to benefit from neoadjuvant immunotherapy remains a significant challenge. While existing biomarkers (such as PD-L1 expression, TMB, and microsatellite instability-high [MSI-H]) have guiding value in some cancer types, their predictive value for NSCLC remains insufficiently validated (37). For example, the SAKK 16/14 study showed no significant correlation between pre-treatment PD-L1 levels and MPR or lymph node downstaging (14). This indicates that single biomarkers are inadequate for accurately reflecting immunotherapy responses, and there is a pressing need for more dynamic, quantifiable, and reproducible composite markers. Future research will likely focus on integrating multidimensional information, including gene expression profiling, APMs, and TIME features. Additionally, emerging biomarkers, such as ctDNA dynamic monitoring, radiomics, gut microbiome composition, and peripheral blood inflammatory markers (e.g., neutrophil-to-lymphocyte ratio [NLR]), hold promise for precise efficacy and prognosis prediction, pushing immunotherapy toward the era of truly personalized treatment.

Second, treatment strategies for driver gene-positive NSCLC patients remain notably underdeveloped. Patients harboring driver mutations such as EGFR mutations or ALK rearrangements are often considered to have “cold” tumors, where the tumor microenvironment is not conducive to the efficacy of ICIs. As a result, these patients are often excluded from key clinical trials (38). Even when some of these patients achieve pathological responses after receiving chemotherapy combined with immunotherapy, their recurrence risk remains significantly higher compared to driver gene-negative populations (39). In response to this issue, the exploration of targeted therapies in the neoadjuvant setting has become a promising direction. For example, osimertinib has shown good short-term efficacy in EGFR-mutant patients, although long-term survival data are still awaited (11). Future research should focus on evaluating the potential of targeted monotherapy, targeted chemotherapy combinations, and targeted immunotherapy combinations to develop personalized treatment regimens for this patient group (38).

Resistance mechanisms also represent a key barrier to long-term benefits from neoadjuvant immunotherapy. Some patients exhibit no response early in treatment (primary resistance), while others show disease progression after initial benefit (acquired resistance) (1). The mechanisms underlying resistance are complex, involving intrinsic tumor factors (such as mutations in β2-microglobulin leading to defects in antigen presentation) as well as immunosuppressive cell infiltration and activation of signaling pathways within the immune microenvironment (7). To address this, rational combination therapies are crucial. Radiation therapy can enhance immune effects by inducing immunogenic cell death, and nanomedicine-based drug delivery can provide targeted delivery and improve drug distribution (40). The integration of these multi-layered approaches may become a key breakthrough in enhancing the durability of treatment effects.

Regarding treatment regimen design, there is currently no unified consensus. Key questions, including the optimal treatment duration, the sequence of immunotherapy and chemotherapy (whether synchronous or sequential), and the necessity and approach to postoperative adjuvant therapy, require further clarification. A retrospective study showed that patients receiving neoadjuvant chemo-immunotherapy followed by postoperative chemo-immunotherapy had significantly better disease-free survival (DFS) and OS compared to those receiving only adjuvant chemotherapy, suggesting that “continuous immunologic management” could offer additional benefits (41). However, for patients who achieve pCR after neoadjuvant therapy, it remains uncertain whether intensified treatment is necessary or if de-escalation strategies should be considered, and this needs further clinical validation. Another study indicated that for some stage III patients, surgery after good response to neoadjuvant therapy resulted in significantly longer progression-free survival (PFS) and OS compared to continuing immune therapy, providing new evidence for treatment decisions in locally advanced patients (42).

Finally, the limitations of efficacy evaluation standards are another important bottleneck in clinical practice. The traditional RECIST criteria primarily rely on changes in tumor volume assessed by imaging, which may not fully reflect the true biological response to immunotherapy. Some patients may experience “pseudoprogression” due to immune cell infiltration, manifesting as temporary tumor enlargement or density changes on imaging, which leads to inconsistencies with pathological results (43). This issue directly impacts surgical timing and efficacy assessment. In the future, functional imaging techniques (such as PET-CT) and AI-assisted radiomic analysis may allow for more precise monitoring and early prediction of treatment efficacy, contributing to the standardization of neoadjuvant immunotherapy application (44).

Overall, the application of neoadjuvant immunotherapy in NSCLC is still evolving. Key research directions include patient selection, resistance mechanisms, regimen optimization, and the refinement of efficacy evaluation systems. Through multidisciplinary collaboration and the integration of basic and translational research, there is potential for more precise and effective neoadjuvant immunotherapy regimens, ultimately leading to more sustained benefits for patients.

## 6 Future prospects

Neoadjuvant immunotherapy for lung cancer is at a rapidly evolving crossroads, with future research set to focus on more precise, personalized, and efficient treatment strategies. While the current most successful model is the combination of immunotherapy and chemotherapy, the side effects associated with chemotherapy cannot be ignored.

Therefore, “chemotherapy-free” regimens represent a critical frontier. Strategies such as dual ICI blockade (e.g., anti-PD-1/L1 plus anti-CTLA-4) or combinations with novel immune agonists (e.g., anti-LAG-3 or anti-TIGIT) are being explored to overcome resistance via distinct checkpoints. Furthermore, integrating immunotherapy with anti-angiogenesis agents or radiation could yield synergistic effects, offering robust alternatives for patients ineligible for chemotherapy (40).

**Figure 2:**
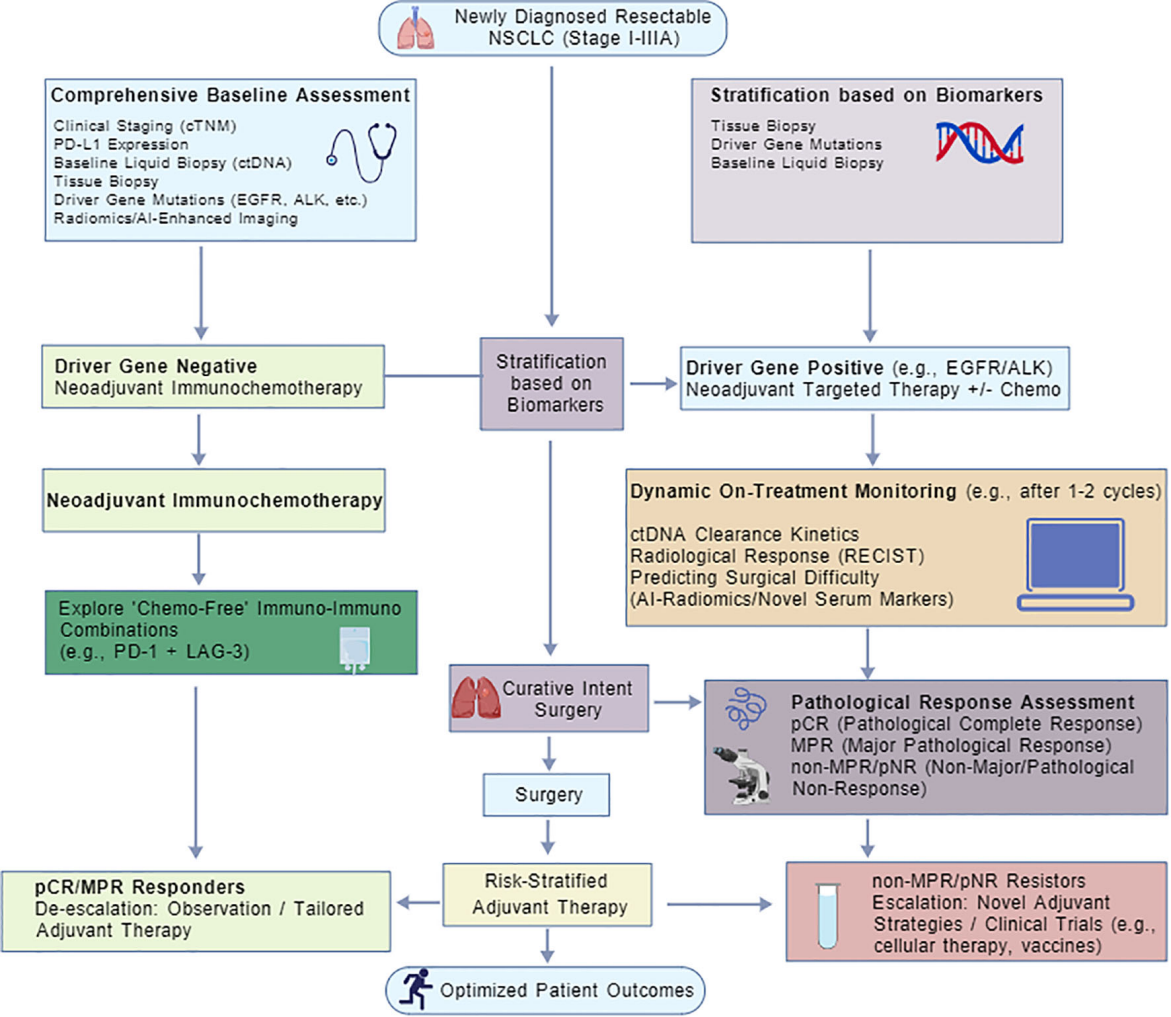
Integrative framework for future personalized therapy. This schematic illustrates a biologically feedback-driven adaptive clinical decision algorithm. It aims to advance the perioperative management of early-stage resectable non-small cell lung cancer (NSCLC) from a traditional “one-size-fits-all” model to a precision medicine paradigm. The management pathway initiates with a comprehensive baseline assessment. By integrating clinical staging, PD-L1 expression, driver mutation status, and baseline characteristics derived from radiomics and liquid biopsy, a multidimensional biological profile is established for each patient. Subsequent therapeutic selection is guided by biomarker stratification: patients negative for driver genes receive standard neoadjuvant chemo-immunotherapy or are considered for “chemo-free” dual immunotherapy regimens; conversely, patients positive for driver genes (e.g., EGFR or ALK mutations) are routed into neoadjuvant targeted therapy pathways. During treatment implementation, dynamic monitoring via ctDNA kinetics and AI-driven radiomics is incorporated to evaluate early molecular responses after 1–2 cycles and predict surgical complexity. Following radical resection, a risk-stratified adjuvant strategy is implemented based on standard pathological response assessment (pCR, MPR, or non-response) combined with molecular residual disease (MRD) status. “Strong responders” achieving deep remission undergo de-escalated observation or personalized dose-reduction therapies, while “resistant patients” exhibiting poor response are escalated to intensified regimens—including cell therapy, tumor vaccines, or novel agents—to optimize long-term prognosis.

As neoadjuvant therapy becomes more widespread, personalized treatment pathways based on pathological responses will serve as an important pivot for achieving precision medicine (**Figure 2**). Risk stratification based on postoperative pathological responses will allow for adaptive treatment strategies: for patients who achieve pCR, it may be possible to simplify or even exempt them from postoperative adjuvant therapy, thus reducing the treatment burden; for patients with poor pathological response (non-MPR), more aggressive postoperative interventions, such as adjusting drug regimens or enrolling in clinical trials, would be necessary (1). This dynamic, adjusted treatment model will propel neoadjuvant immunotherapy toward true individualization and precision.

The development of innovative technologies will further empower this field. Liquid biopsy, particularly the dynamic monitoring of ctDNA, is expected to enable real-time, non-invasive assessment of treatment efficacy and even predict the onset of resistance (37). Artificial intelligence (AI) will also play a crucial role, from automatically quantifying responses in pathological images to extracting radiomic features from imaging and integrating multi-omics data (genomics, transcriptomics, proteomics) to create predictive models for individualized treatment decisions. Meanwhile, advances in nanotechnology will enable drug co-delivery, targeted release, and microenvironment-responsive treatments, improving efficacy while minimizing toxicity, offering new ideas for designing future therapeutic platforms (44).

In addition to optimizing existing strategies, exploring entirely new immunotherapy models is also crucial. Therapeutic cancer vaccines, including peptide, mRNA, and dendritic cell vaccines, hold promise for inducing more durable anti-tumor immune responses by introducing specific antigens. When combined with ICIs, these vaccines may achieve synergistic effects (i.e., “1 + 1>2”). Furthermore, cell therapies such as CAR-T and TCR-T, although still in the early stages of research in solid tumors, hold great potential for eradicating micro-metastases as technological breakthroughs occur (7).

Overall, neoadjuvant immunotherapy has become a transformative development in the treatment of resectable NSCLC (45). Numerous clinical trial results, including the CheckMate-816 study, have established the value of immune combination chemotherapy, making it the new standard of care (3). However, it is important to recognize that this field is still in its early stages. Precision biomarker screening, optimization of strategies for driver gene-positive populations, elucidation and overcoming of resistance mechanisms, and the refinement of treatment management remain significant challenges that need to be addressed in the future. Behind each of these challenges lies new opportunities, pushing us toward more personalized and effective treatments.

The future development will be multidimensional, exploring both “chemotherapy-free” and novel combination strategies, as well as clinical applications of technologies such as liquid biopsy and artificial intelligence, and extending to clinical translation of cancer vaccines and cell therapies. To achieve these goals, close collaboration between basic research, translational medicine, and clinical trials, along with cooperative efforts from multidisciplinary teams, is essential. As research progresses and practical experience accumulates, neoadjuvant immunotherapy for lung cancer is expected to continue driving innovation in the treatment paradigms for early-stage lung cancer, ultimately offering hope for long-term survival and even cure for more patients.

## Data Availability

The original contributions presented in the study are included in the article/supplementary material, further inquiries can be directed to the corresponding author.
